# Single-crystal Raman spectroscopy and X-ray crystallography at beamline X26-C of the NSLS

**DOI:** 10.1107/S0909049510033601

**Published:** 2010-11-05

**Authors:** Deborah Stoner-Ma, John M. Skinner, Dieter K. Schneider, Matt Cowan, Robert M. Sweet, Allen M. Orville

**Affiliations:** a Biology Department, Brookhaven National Laboratory, Upton, NY 11973, USA

**Keywords:** Raman, single-crystal spectroscopy, X-ray diffraction

## Abstract

Three-dimensional structures derived from X-ray diffraction of protein crystals provide a wealth of information. Features and interactions important for the function of macromolecules can be deduced and catalytic mechanisms postulated. Still, many questions can remain, for example regarding metal oxidation states and the interpretation of ‘mystery density’, *i.e.* ambiguous or unknown features within the electron density maps, especially at ∼2 Å resolutions typical of most macromolecular structures. Beamline X26-C at the National Synchrotron Light Source (NSLS), Brookhaven National Laboratory (BNL), provides researchers with the opportunity to not only determine the atomic structure of their samples but also to explore the electronic and vibrational characteristics of the sample before, during and after X-ray diffraction data collection. When samples are maintained under cryo-conditions, an opportunity to promote and follow photochemical reactions *in situ* as a function of X-ray exposure is also provided. Plans are in place to further expand the capabilities at beamline X26-C and to develop beamlines at NSLS-II, currently under construction at BNL, which will provide users access to a wide array of complementary spectroscopic methods in addition to high-quality X-ray diffraction data.

## Abstract 2

The collection of absorption and Raman spectroscopic data correlated with X-ray diffraction data allows investigators to understand the atomic structure as well as the electronic and vibrational characteristics of their samples, to identify transiently formed intermediates and to explore mechanistic questions. Raman spectroscopy instrumentation at beamline X26-C at the NSLS is currently available to the general user population.

## 1. Introduction

Understanding the complex relationships among atomic structure, electronic structure and chemistry is crucial for obtaining insights into the fundamental biological processes including those that (*a*) underlie human diseases and (*b*) can contribute to the development of clean energy sources. A variety of spectroscopic methods can provide a means with which to explore these relationships, particularly when the sample includes a conjugated cofactor or ligand. Indeed, approximately one-third of all macromolecules expressed in organisms contain a non-protein cofactor (Waldron *et al.*, 2009 ▶). Cofactors with color, such as metal ions and/or organic molecules, are present in approximately 11000 entries in the Protein Data Bank archive as of June 2010. These macromolecules usually exhibit a characteristic optical absorption spectrum that often changes during catalysis and/or during X-ray diffraction data collection. Post-translational modifications can also result in unique spectroscopic characteristics. Spectroscopic studies are also useful for proteins co-crystallized with substrate or product analogs (De la Mora-Rey & Wilmot, 2007 ▶).

To conduct concurrent studies utilizing both crystallographic and spectroscopic methods, an integrated infrastructure is required at the X-ray beamline. Such a facility has been developed at beamline X26-C of the National Synchrotron Light Source (NSLS) and other synchrotrons (Owen *et al.*, 2009 ▶; McGeehan *et al.*, 2009 ▶; Davies *et al.*, 2009 ▶; Ellis *et al.*, 2008 ▶; Royant *et al.*, 2007 ▶; Carpentier *et al.*, 2007 ▶). Beamline X26-C is currently capable of following changes in the visible absorption spectrum and in the vibrational spectrum *via* Raman spectroscopy as a function of X-ray exposure. The design and use of the recently added Raman system is presented herein; a separate article will present the design and implementation of correlated absorption spectroscopy with X-ray diffraction (Orville *et al.*, 2011 ▶).

## 2. Materials and methods

### 2.1. Instrumentation

The facility at beamline X26-C at NSLS supports correlated X-ray diffraction and spectroscopic analysis of macromolecules, utilizing specifically visible absorption spectroscopy and Raman vibrational spectroscopy, each at a 90° orientation to the X-ray beam. The instrumentation and methodology for single-crystal visible absorption spectroscopy has already been described (Héroux *et al.*, 2009 ▶; Orville *et al.*, 2009 ▶, 2011 ▶; Yi *et al.*, 2010 ▶).

**Figure 1:**
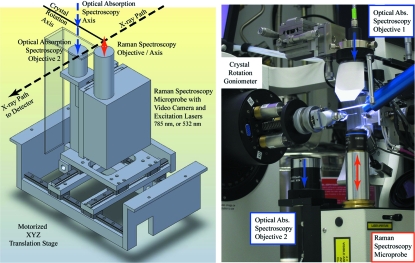
The current configuration at beamline X26-C for correlated X-ray diffraction, electronic absorption and Raman spectroscopy. The left-hand image is a schematic of the *x*, *y*, *z* translation stage supporting the Raman SuperHead and absorption mode collection objective. The motorized stage allows the user to easily switch between absorption and Raman data collection modes in less than 60 s. The image on the right illustrates a close-up view around the sample in an alignment mode for Raman spectroscopy. The direction of the X-ray beam projects out of the page.

In this report we focus on Raman spectroscopy. We currently collect off-resonance Raman spectra utilizing a frequency-stabilized multimode 785 nm diode laser (Process Instruments, Salt Lake City, UT, USA) and near-resonance Raman spectra with a diode-pumped solid-state single longitudinal-mode 532 nm laser (Laser Quantum, Cheshire, UK). Laser line widths are 30 GHz (multimode) and 1 MHz, respectively. The beam is delivered *via* a 100 µm-diameter fiber optic to a wavelength-specific Horiba Jobin-Yvon (Edison, NJ, USA) Raman SuperHead. The SuperHead contains an edge filter to both select the excitation wavelength and to remove that wavelength from the collected scattered signal. An Olympus SLMPlanN 50×/0.35 objective is used to focus the beam to a ∼25 µm spot size. The focal depth, *f*, is 3.6 µm. The region of the crystal probed is the same as that used for both X-ray diffraction and absorption data collection, as will be discussed below. The Raman SuperHead and the collection objective for absorbance mode are located below the crystal on an *x*, *y* and *z* translation stage (Fig. 1 ▶). This motorized stage allows for rapid switching between alignments optimized for electronic absorption and for Raman spectroscopy. Reproducibility of the stage position was determined by the manufacturer (CrystalLogic, CA, USA) to be within 2.5 µm in each of *x*, *y* and *z*. The SuperHead collects both Rayleigh and Raman scattered light in a backscatter (180°) mode. An edge filter within the SuperHead removes the Rayleigh scatter. The Raman scattered light is focused into another 100 µm fiber optic which connects to an iHR320 spectrometer (Horiba Jobin-Yvon). The entrance slit width is adjustable with a maximal width of 2 mm. Three gratings on a motorized turret are available: 600, 1200 and 1800 lines mm^−1^. The user can easily switch between the collection of broad low-resolution spectra and the collection of high-resolution spectra from a narrow region of particular interest. The detector is a Peltier-cooled (203 K) Synapse CCD detector (Horiba Jobin-Yvon). The CCD chip is a front-illuminated open electrode 1024 × 256 pixel array with a 26 × 26 µm pixel size. Laser calibration is assured using a Ne calibration lamp (Oriel, Stratford, CT, USA) or fluorescent room light. Overall instrument resolution using the 1200 grating is 4 cm^−1^ (Horiba specifications).

Of vital importance to the success of correlated studies is the co-localization of the crystal rotation axis, the X-ray beam and the focal point of each spectroscopic method such that an identical region of the crystal is probed. Two cameras are used for alignment. One camera views the crystal from 35° below the X-ray path and is used for centering the crystal to the X-ray beam. This camera is also used to view the alignment of the focal points of the spectroscopy objectives on a 400/10 micromesh (MiteGen, Ithaca, NY, USA). A second line-of-sight camera is contained within each of the Raman SuperHeads and facilitates aligning the Raman lasers to the crystal eucentric rotation point and the X-ray beam. Exposure of the mesh to the laser must be minimized to prevent melting of the mesh; typical exposure times are brief (<10 s) with the laser attenuated to deliver 1 mW.

### 2.2. Software integration

Software control for absorption spectroscopy has been fully incorporated into the beamline control software (Yi *et al.*, 2010 ▶; Orville *et al.*, 2011 ▶). Raman data acquisition is currently controlled by *LabSpec* 5.55.10, a Windows-based software provided by Horiba Jobin-Yvon. Future plans include transferring control of data acquisition to the beamline control software in a manner similar to that done for absorption spectroscopy.

### 2.3. Data collection

After the crystal is centered for X-ray diffraction collection, the crystal orientation providing the optimal Raman spectrum is typically determined manually by collecting 10 s spectra at approximately 10° increments of rotation, with the laser attenuated such that only the strongest Raman vibration modes are observed. This is to avoid laser-induced damage to the crystal. Crystal orientations that result in the lowest cleanest background and the best signal-to-noise ratio are identified. Power levels and exposure times also have to be optimized for each sample, as tolerance to the laser will vary from sample to sample. Typical conditions for a full protein Raman spectrum acquisition include total collection times of 1 to 5 min and laser powers of 2–10 mW at the sample.

## 3. Results and discussion

### 3.1. Preliminary Raman experiments

#### 3.1.1. Zn^2+^ insulin

**Figure 2:**
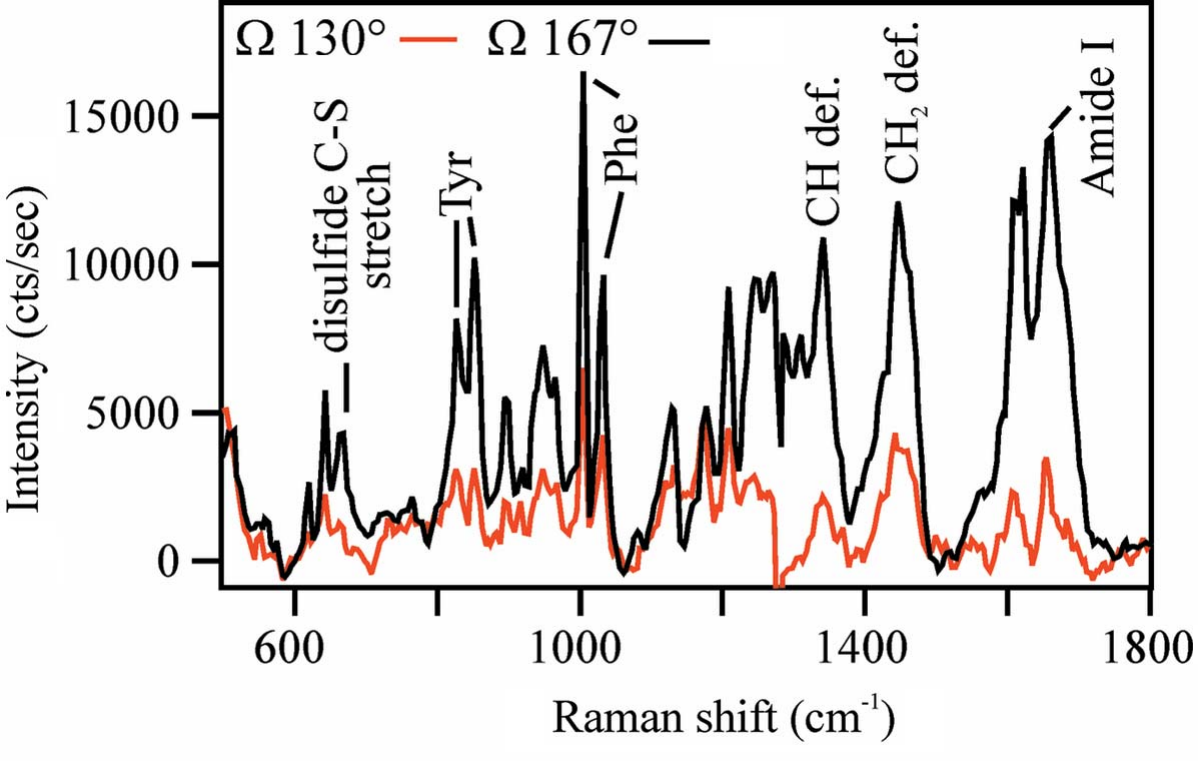
Raman spectra of a Zn^2+^ insulin crystal. Raman spectra were taken at Ω = 130° (red) and 167° (black) where Ω = 0° is defined as one of the angles at which the flat face of the crystal is orthogonal to the Raman objective. The spectra were collected using 6 mW of 532 nm laser excitation and 50 s total acquisition time. Peaks assignments are based on Yu *et al.* (1972 ▶).

Use of the Raman system at X26-C was inaugurated during the April 2010 RapiData crystallography course run by the Macromolecular Crystallography Research Resource (PXRR) at BNL. Raman spectra using the 532 nm laser source were collected on several protein crystals, including a Zn^2+^ insulin crystal provided by the course (Soares *et al.*, 2003 ▶). The importance of investigating the crystal orientation angle (Ω) relative to the X-ray beam axis was immediately recognized from the insulin spectra taken at the two angles, 130° and 167° (Fig. 2 ▶). Both the intensity and quality of the spectra varied greatly. We and others have suggested that anisotropic absorption and the prism effects of the single crystal impact electronic absorption and Raman scattering (Carey, 2006 ▶; Pearson *et al.*, 2007 ▶). If a planar chromophore is present within the crystal, that too impacts the intensity of Raman scattering of individual modes (Stoner-Ma *et al.*, 2006 ▶). It is also important to reduce the likelihood of artefacts inherent to the sample mount; for example, the nylon loop should not intersect the Raman laser beam path. To prevent loop contributions, we often collect absorption data with the flat face of the loop perpendicular to the spectroscopy beam. This orientation also frequently presents the broadest face of the crystal to the beam. The optimal orientation for Raman spectroscopy is not necessarily the same as that for absorption spectroscopy. In the case of Zn^2+^ insulin, the approximate perpendicular orientation was when Ω was set to 105°. However, a better Raman spectrum was collected at an Ω angle of 167°, *i.e.* closer to a parallel than perpendicular orientation of the loop to the laser beam. Comparison of the obtained Zn^2+^ insulin spectrum with a published Raman spectrum of insulin (Yu *et al.*, 1972 ▶) allows assignment of several of the normal modes, as indicated in Fig. 2 ▶. The quality of the spectrum obtained from a protein such as insulin, which lacks a chromophore, indicates that X-ray-induced vibrational changes, if they occur, may be discernible by non-resonant Raman spectroscopy. An example might include the breakage of a disulfide bond and concomitant loss of the S—S mode at approximately 510 cm^−1^. Non-resonant Raman spectroscopy has already been used to follow X-ray damage in brominated DNA (McGeehan *et al.*, 2007 ▶). Intensity changes of a carbon–bromine mode were noted as well as some normal modes shifts of 10–17 cm^−1^. In protein samples, changes of this magnitude may also be observed. An example would be shifts owing to changes in hydrogen bonding or salt bridges within the protein. Assignment of the normal modes responsible for changes observed in proteins would likely require theoretical calculations, difference Raman spectroscopy and/or isotope editing.

#### 3.1.2. Orientation dependence

**Figure 3:**
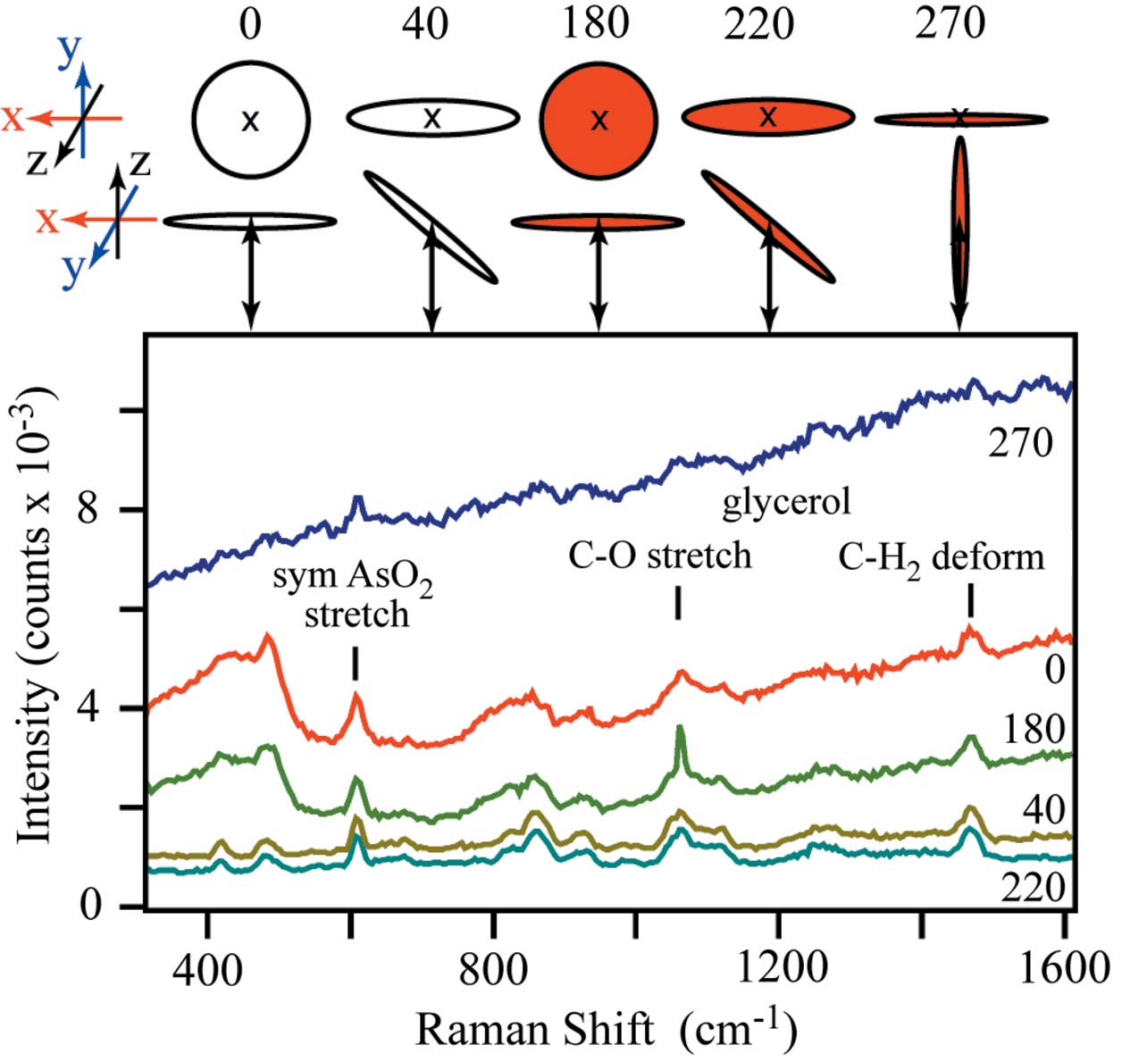
Raman spectra of frozen 50% glycerol, 0.5 *M* cacodylate pH 8 using 2.6 mW 532 nm laser excitation and a 10 s collection time. Spectra were collected every 10° of loop rotation about the omega axis (Ω). Selected spectra are shown to illustrate the dependence of spectral quality on Ω: 0° (red), 40° (olive), 180° (green), 220° (light blue) and 270° (dark blue). The top of the figure indicates loop orientation at each Ω, with the red and white color scheme distinguishing the two flat faces. The right-handed coordinate system at beamline X26-C has the X-ray beam travelling along *x*, the crystal rotation axis parallel to *y*, and the Raman spectroscopy axis along *z*. The 600 lines mm^−1^ grating and a 1 mm spectrometer entrance slit size were used. The assignment of the symmetric AsO_2_ stretch of cacodylate is from Thuy *et al.* (2010 ▶); those for glycerol are from Mendelovici *et al.* (2000 ▶).

The role of sample orientation relative to the laser beam has been examined further using a frozen glass of a buffer and cryo-protectant commonly used in macromolecular crystallography. A loop holding a cryo-cooled thin film (approximately 15 µm) of 50% glycerol (*v*/*v*) and 0.5 *M* sodium cacodylate pH 8 was oriented such that when the loop Ω angle was set to 0° a flat side of the loop was orthogonal to the laser beam. The resulting spectra showed great variation in the intensity of the glycerol C—O stretch, glycerol CH_2_ deformation and AsO_2_ symmetric stretch modes as Ω is changed (Fig. 3 ▶). While, again, it might be assumed that orienting the flat face of the sample orthogonal to the laser beam (*i.e.* 0° and 180° Ω) would yield the best spectra, this is clearly not the case. The 0° and 180° Ω spectra do contain the strong glycerol and cacodylate modes, but they also exhibit a high background signal, especially in the 400–500 cm^−1^ region. The higher background may be a result of prism effects of the sample. The intense and broad 400–500 cm^−1^ feature has been observed in spectra from other samples, including protein crystals, and may serve as an indicator of an un-optimized Ω. Both spectra in which the loop is edge-on (*i.e.* 90° and 270° Ω) to the laser beam show very high backgrounds with only barely identifiable vibrational modes from the sample. Contributors to these results include an un-optimized focal point and contributions or reflections from the loop itself. The 270° Ω spectrum is included in Fig. 3 ▶; the 90° spectrum is not shown due to saturation of the detector. The best spectrum in terms of low background and strong sample peaks was obtained at Ω = 220°. Rotating the sample 180° further to Ω = 40° also yielded a clean spectrum. For this specimen, therefore, these are the angles of choice for the Raman spectroscopy, and not the Ω angles of 0° and 180° as one might easily assume. Similar results have been observed for several protein crystals in which the optimal angle for Raman collection was not face on (*vide infra*). With protein crystals, there will be additional contributions to the Raman spectra, including the orientation-dependent sample volume probed and possible fluorescence. Our results indicate that assumptions cannot be made as to the optimal Raman orientation but needs to be determined for each and every sample.

### 3.2. Current studies

Collaborative studies incorporating Raman collection together with absorption and diffraction data are now underway. Results from studies on a Rieske iron–sulfur protein have shown specific vibrational mode shifts following X-ray exposure, clearly indicating a rapid reduction upon X-ray exposure. These results will be published shortly. Similar experiments with a variety of other iron-containing proteins are planned. Studies will be continued on the choline oxidase system in which a FAD C4a-oOH or C4a-OOH adduct had been identified using absorption spectroscopy and X-ray diffraction structural data (Orville *et al.*, 2009 ▶).

Moving forward, we will continue to expand the capabilities at X26-C to include fluorescence (steady-state and time-resolved) spectroscopy and to provide a wider selection of excitation wavelengths for Raman spectroscopy (633 and 442 nm lasers are currently planned). Controls for Raman acquisition will be fully integrated into the beamline control software, as has been done for absorption spectroscopy. A complementary off-line spectroscopy facility is under construction immediately adjacent to the beamline. Lastly, with the completion of NSLS-II expected in 2015, a proposal has been submitted for a fully integrated spectroscopy-enhanced X-ray beamline. This beamline, if approved and funded, would maximally exploit the potential of this new third-generation 3 GeV synchrotron.

## References

[bb1] Carey, P. R. (2006). *Annu. Rev. Phys. Chem.* **57**, 527–554. 10.1146/annurev.physchem.57.032905.104521 16599820

[bb2] Carpentier, P., Royant, A., Ohana, J. & Bourgeois, D. (2007). *J. Appl. Cryst.* **40**, 1113–1122.

[bb3] Davies, R. J., Burghammer, M. & Riekel, C. (2009). *J. Synchrotron Rad.* **16**, 22–29. 10.1107/S0909049508034663 19096170

[bb4] De la Mora-Rey, T. & Wilmot, C. M. (2007). *Curr. Opin. Struct. Biol.* **17**, 580–586. 10.1016/j.sbi.2007.09.005 PMC2134968 17959373

[bb5] Ellis, M. J., Buffey, S. G., Hough, M. A. & Hasnain, S. S. (2008). *J. Synchrotron Rad.* **15**, 433–439. 10.1107/S0909049508014945 18728313

[bb6] Héroux, A., Bozinovski, D. M., Valley, M. P., Fitzpatrick, P. F. & Orville, A. M. (2009). *Biochemistry*, **48**, 3407–3416. 10.1021/bi8023042 PMC2754721 19265437

[bb7] McGeehan, J. E., Carpentier, P., Royant, A., Bourgeois, D. & Ravelli, R. B. G. (2007). *J. Synchrotron Rad.* **14**, 99–108. 10.1107/S0909049506043251 17211076

[bb8] McGeehan, J., Ravelli, R. B. G., Murray, J. W., Owen, R. L., Cipriani, F., McSweeney, S., Weik, M. & Garman, E. F. (2009). *J. Synchrotron Rad.* **16**, 163–172. 10.1107/S0909049509001629 PMC2651762 19240328

[bb10] Mendelovici, E., Frost, R. L. & Kloprogge, T. (2000). *J. Raman Spectrosc.* **31**, 1121–1126.

[bb11] Orville, A. M., Lountos, G. T., Finnegan, S., Gadda, G. & Prabhakar, R. (2009). *Biochemistry*, **48**, 720–728. 10.1021/bi801918u PMC2646362 19133805

[bb12] Orville, A. M., Stoner-Ma, D., Skinner, J. M., Héroux, A., Schneider, D. K. & Sweet, R. M. (2011). *J. Synchrotron Rad.* In preparation.

[bb13] Owen, R. L., Pearson, A. R., Meents, A., Boehler, P., Thominet, V. & Schulze-Briese, C. (2009). *J. Synchrotron Rad.* **16**, 173–182. 10.1107/S0909049508040120 PMC2651763 19240329

[bb14] Pearson, A. R., Pahl, R., Kovaleva, E. G., Davidson, V. L. & Wilmot, C. M. (2007). *J. Synchrotron Rad.* **14**, 92–98. 10.1107/S0909049506051259 17211075

[bb15] Royant, A., Carpentier, P., Ohana, J., McGeehan, J., Paetzold, B., Noirclerc-Savoye, M., Vernède, X., Adam, V. & Bourgeois, D. (2007). *J. Appl. Cryst.* **40**, 1105–1112.

[bb16] Soares, A. S., Caspar, D. L. D., Weckert, E., Héroux, A., Hölzer, K., Schroer, K., Zellner, J., Schneider, D., Nolan, W. & Sweet, R. M. (2003). *Acta Cryst.* D**59**, 1716–1724. 10.1107/s0907444903015403 14501109

[bb17] Stoner-Ma, D., Melief, E. H., Nappa, J., Ronayne, K. L., Tonge, P. J. & Meech, S. R. (2006). *J. Phys. Chem. B*, **110**, 22009–22018. 10.1021/jp065326u 17064171

[bb18] Thuy, N. T. B., Yokogawa, R., Yoshimura, Y., Fujimoto, K., Koyan, M. & Maenosono, S. (2010). *Analyst*, **135**, 595–602. 10.1039/b919969a 20174716

[bb19] Waldron, K. J., Rutherford, J. C., Ford, D. & Robinson, N. J. (2009). *Nature (London)*, **460**, 823–830. 10.1038/nature08300 19675642

[bb20] Yi, J., Orville, A. M., Skinner, J., Skinner, M. & Richter-Addo, G. B. (2010). *Biochemistry*, 49, 5969–5971. 10.1021/bi100801g PMC2916933 20568729

[bb21] Yu, N.-T., Liu, C. S., Culver, J. & O’Shea, D. C. (1972). *Biochim. Biophys. Acta*, **263**, 1–6. 10.1016/0005-2795(72)90153-5 5062505

